# 

**Published:** 1920-12-18

**Authors:** 


### Matrons' and Sisters' Appointments.

Aberdare and District General Hospital.—Miss Ethel Richards has been appointed sister. She was trained at the Township Infirmary, Leeds, and has been staff nurse at the War Pensions Hospital, Birmingham, sister at the Auxiliary Military Hospital, Griffithstown, and ward sister at Bromsgrove Cottage Hospital.

Royal Infirmary, Sheffield.—Miss Flora M. Hill has been appointed home sister. She was trained at the Middlesex Hospital, the London Fever Hospital, and at Her Majesty's Hospital, Stepney Causeway. She holds the certificate of the Central Midwives Board, and for some time has been theatre sister at the Royal Infirmary, Bradford.

Borough Hospital, Burton-on-Trent.—Miss Gwladys Williams has been appointed sister. She was trained at the Royal Infirmary, Manchester, and at Ladywell Sanatorium, Salford, at which latter institution she has been staff nurse and sister.

Victoria Hospital, Southend-on-Sea.—Miss D. Gibson has been appointed sister. She was trained at the Middlesex Hospital and the Evelina Hospital for Sick Children. She has been temporary sister at the former hospital, and has seen five years' service under Queen Alexandra's Imperial Military Nursing Service Reserve. She is a member of the College of Nursing.

Kensington Infirmary.—Miss Dora Manning has been appointed superintendent night nurse. She was trained at Kensington Infirmary, where she has since been ward sister.

Tunbridge Wells General Hospital.—Miss Catherine M. Speed has been appointed sister. She was trained at Leicester Royal Infirmary, where she has since been theatre charge nurse.

General Nursing Council.—Miss G. E. Davies has been appointed registration clerk. She was trained at the Royal Southern Hospital, Liverpool, and has since been sister of the women's medical and surgical wards and night sister at the Royal Hospital, Richmond; nursing sister in France with St. John Ambulance Association and with the Serbian Relief Fund in Serbia. She has also been sister-in-charge of the out-patient department at the South London Hospital for Women.

